# A Middle‐Aged Man With Pulseless VT and Dual Pathology: Anomalous Left Main Coronary Artery From Right Coronary Cusp With Transseptal Course and Underlying Dilated Cardiomyopathy

**DOI:** 10.1002/ccr3.71408

**Published:** 2025-11-04

**Authors:** Amir Heidari, Enssieh Hashemi, Mehrdad Jafari Fesharaki, Golnaz Houshmand, Roghaye Ghiasvand‐Mohammadkhani

**Affiliations:** ^1^ Department of Cardiology, Imam Hossein Hospital Shahid Beheshti University of Medical Sciences Tehran Iran; ^2^ Department of Neurology, Fatemeh Zahra Hospital Iran University of Medical Sciences Tehran Iran; ^3^ Department of Cardiology, School of Medicine Shahid Beheshti University of Medical Sciences Tehran Iran; ^4^ Cardiovascular Imaging Reseach Centre Rajaie Cardiovascular Institute Tehran Iran; ^5^ Shahid Beheshti University of Medical Sciences Tehran Iran

**Keywords:** anomalous left main coronary artery, cardiac arrest, dilated cardiomyopathy, transseptal course, ventricular tachycardia

## Abstract

Anomalous aortic origin of a coronary artery from the opposite sinus (AOCAOS) is a rare variety of coronary artery anomalies. Left main coronary artery (LMCA) arising from right coronary cusp (RCC) with a transseptal course is an uncommon variant that may not be as benign as previously thought. The coexistence of AOCAOS with dilated cardiomyopathy (DCM) is exceptionally rare and presents diagnostic and therapeutic challenges. Herein, we present a 43‐year‐old man with a history of Type 2 diabetes mellitus and chronic substance use who was admitted after successful resuscitation from pulseless ventricular tachycardia (VT). Initial workup revealed severely reduced left ventricular ejection fraction (LVEF) of 20% on echocardiography. Invasive coronary angiography demonstrated anomalous origin of the LMCA from the RCC. Cardiac magnetic resonance (CMR) confirmed the diagnosis of DCM with no evidence of fibrosis, and coronary computed tomography angiography (CCTA) delineated a transseptal course of the anomalous LMCA. The patient was managed medically for heart failure and underwent cardiac resynchronization therapy defibrillator (CRT‐D) implantation for primary prevention of sudden cardiac death. At 3‐month follow‐up, he showed significant clinical improvement. This case underscores the importance of a comprehensive diagnostic approach in sudden cardiac death survivors and highlights that transseptal AOCAOS may not be benign when associated with cardiomyopathy. Coronary CTA and CMR are essential tools for complete evaluation.

**Summary**

- The transseptal course of an anomalous coronary artery, often considered benign, can be associated with life‐threatening arrhythmias and may coexist with cardiomyopathy.
- A comprehensive workup including coronary CTA and CMR is essential when this anomaly is detected to guide optimal management, which may include device therapy for associated cardiomyopathy.

## 1 Introduction

Although coronary anomaly has a low rate of incidence, it is considered the second most common cause of sudden cardiac death (SCD) in young patients [1, 2]. So far, guidelines have not reported any specific recommendation for the diagnosis, workup and treatment of patients with AOCAOS, which makes this disease a medical challenge [1, 3]. A rare form of AOCAOS is anomalous left main coronary artery (LMCA) originating from RCC, which has an incidence rate of 0.02%–0.08% [4] and is the most critical form [5]. In this anomaly, LMCA may pass through different routes to reach its myocardial territory. These routes are classified into four groups: prepulmonic, interatrial, transseptal and retroaortic [6]. The transseptal course of anomalous LMCA is a rare type that used to be considered benign. Still, recent case reports have shown that it could cause life‐threatening cardiac manifestations such as myocardial infarction (MI), arrhythmia and even SCD [7]. The coexistence of AOCAOS with dilated cardiomyopathy (DCM) is exceptionally rare, with only a handful of cases reported in the literature. We present this case to highlight the diagnostic challenge, explore the potential association between these two pathologies, and discuss the therapeutic approach in a patient with this rare dual diagnosis.

## 2 Case Presentation

A 43‐year‐old man with Type 2 diabetes was brought to the ED intubated and unconscious after a sudden pulseless collapse. Bystander BLS was initiated. EMS found and defibrillated pulseless VT, restoring sinus rhythm. On admission, he was stable but unconscious. ECG showed a LBBB with negative Troponin‐I; BNP was elevated at 850 pg/mL. He gradually regained consciousness and was extubated after 12 h. History revealed NYHA class II dyspnea, and chronic use of tobacco, opium, and methadone. He denied angina. Examination revealed an S3 gallop and bilateral basal crackles.

## 3 Methods

Through our diagnostic approach, first, transthoracic echocardiography (TTE) was done, which revealed an LV ejection fraction of 20% with global hypokinesia, moderate left ventricle (LV) enlargement, with no significant valvular heart disease (Video 1).

**VIDEO 1:** Four chamber view of transthoracic echocardiography, which revealed LV ejection fraction of 20% with global hypokinesia, moderate left ventricle (LV) enlargement. Video content can be viewed at https://onlinelibrary.wiley.com/doi/10.1002/ccr3.71408.

**FIGURE 1:**
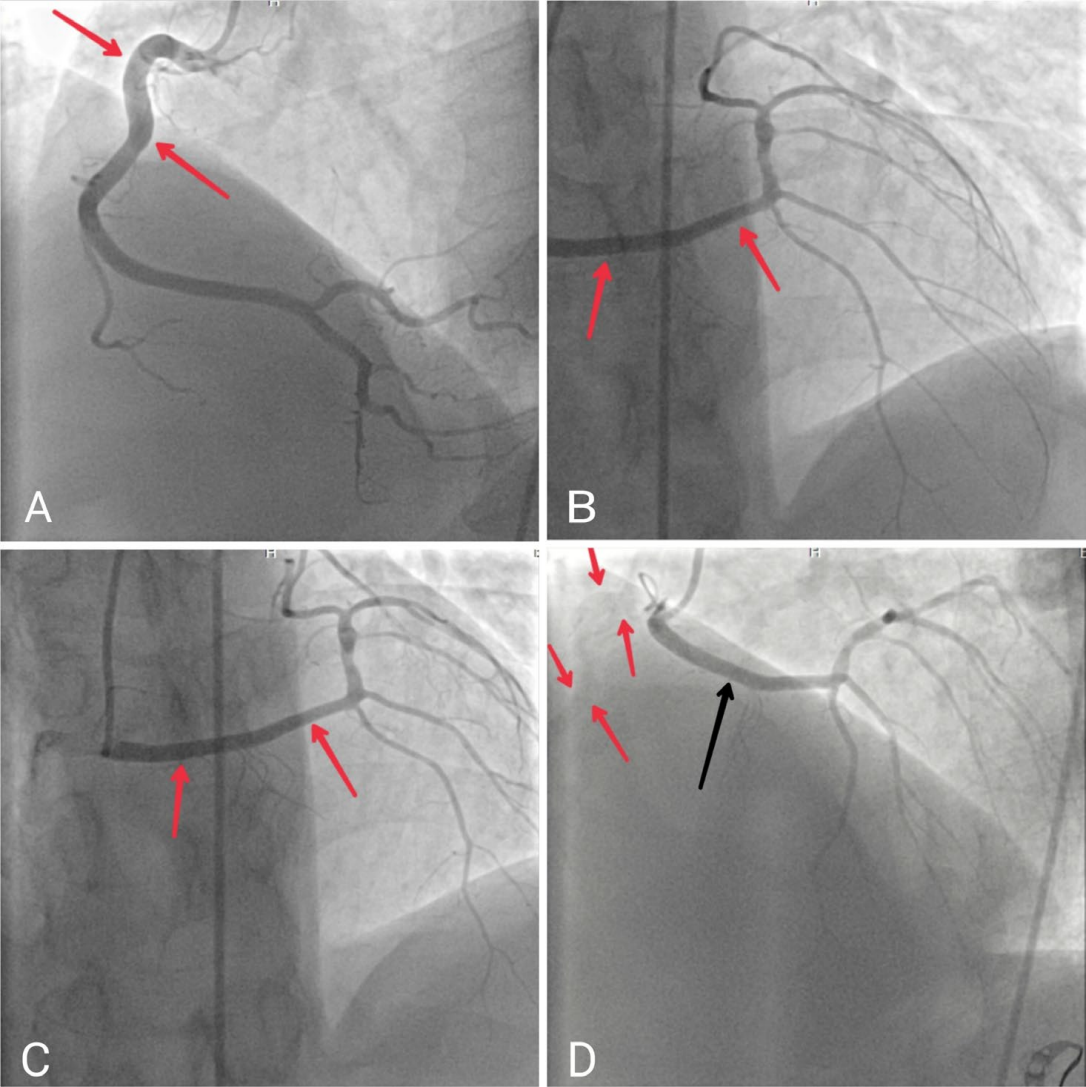
Different views of invasive coronary angiography: (A) The left anterior oblique view of invasive coronary angiography shows the right main coronary artery (red arrows). (B) The anterior posterior cranial view of invasive coronary angiography shows the left main coronary artery (red arrows). (C) The anterior posterior view of invasive coronary angiography shows the left main coronary artery (red arrows). (D) The left anterior oblique view of invasive coronary angiography shows the left main coronary artery (black arrow) and the right coronary artery and its course (red arrows) originating from the right coronary cusp.

Due to low EF, we performed an invasive coronary angiography (ICA) to rule out coronary artery disease. The coronary system was right dominant. Surprisingly, it showed that the ostium of LMCA originated from RCC. There was mild stenosis at the proximal of LCX and moderate stenosis at the ostium of PLV. LAD had no significant plaque (Figure 1).

**FIGURE 2:**
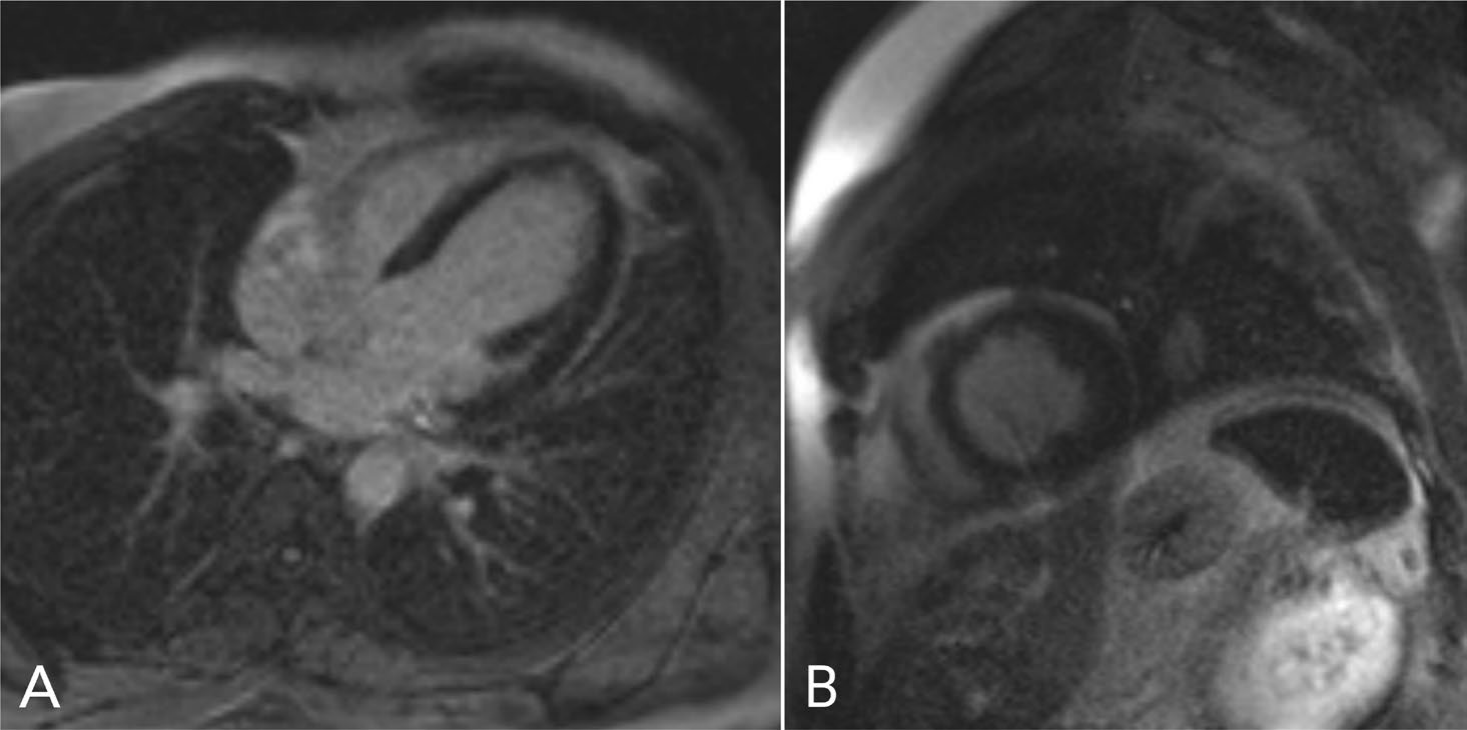
Cardiac magnetic resonance imaging: (A, B) Late gadolinium enhancement images midlevel short axis and four‐chambers show no significant myocardial infarction or fibrosis.

Given that the coronary artery evaluation did not justify the drop in ejection fraction, the patient underwent a cardiac magnetic resonance (CMR) that confirmed severely reduced systolic function, global hypokinesia of LV, moderately enlarged LV size without hypertrophy, and no late gadolinium enhancement (LGE). The diagnosis of dilated cardiomyopathy (DCM) was established as the cause of reduced ejection fraction (Figure 2).

**FIGURE 3:**
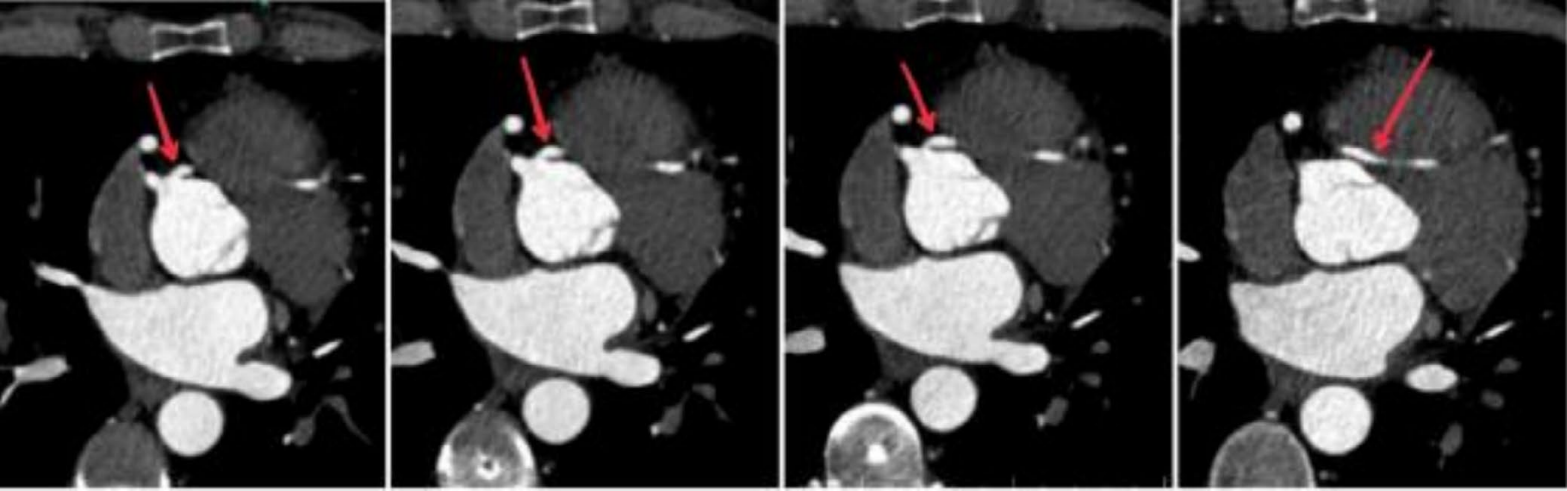
Coronary computed tomography angiography: It shows the origin of the left main coronary artery and its transseptal course.

Considering the aberrant origin of the left main coronary artery from RCC, the patient underwent coronary CT angiography to evaluate the course of the coronary artery, which confirmed the LMCA origin from RCC and also showed a transseptal course of the left main artery (Figure 3).

## 4 Management and Follow‐Up

The patient's reduced EF prompted the initiation of guideline‐directed medical therapy including beta blocker, sacubitril/valsartan, empagliflozin and mineralocorticoid receptor antagonist. Due to the history of VT and the probability of arrhythmia alongside the low EF and his coronary anomaly he underwent implantation of a cardiac resynchronization therapy defibrillator (CRT‐D) based on the patient's LBBB and sinus rhythm on baseline ECG.

After a successful procedure of implanting CRT‐D, the patient was discharged after a 7‐day hospitalization with no signs or symptoms. At a 3‐month follow‐up, the patient reported significant improvement in dyspnea (NYHA Class I), and a repeat echocardiogram showed a modest improvement in LVEF to 25%.

Given the transseptal (non‐interarterial) course of the anomalous LMCA—a variant not typically associated with the same high‐risk features as interarterial courses—and the absence of objective evidence of ischemia directly attributable to the anomaly, a heart team decision was made to manage the patient medically and with device therapy, focusing on the DCM as the primary pathology. However, the patient will be closely followed up and re‐evaluated for any potential need for surgical intervention in the future.

## 5 Discussion

Coronary artery anomalies (CAAs) are rare congenital defects in various forms, such as main branches arising from a wrong sinus of Valsalva, pulmonary artery or a branch of another coronary artery [8]. AOCAOS is a CAA subgroup with an incidence of 0.28%–1.74% [2]. The most common variants are the RCA originating from the left coronary sinus and the LMCA originating from RCC, which have different courses through their routes. Clinical significance and potential complications depend on the ectopic artery's course [6].

The transseptal course of the coronary artery is associated with anomalous origin of LMCA or LAD from the RCC or as a branch of the single coronary artery from RCC. In this course, the artery takes a downward path after its origination, then turns left and continues its route below the aortic and pulmonary annulus level in the posterior wall of the right ventricle outflow tract [7]. In this type, there is a dynamic compression on the artery during the cardiac cycle, leading to reduced blood flow during systole [9], as was likely present in our case.

High‐risk AOCAOS variants, particularly those with an interarterial course, are characterized by features such as a slit‐like ostium, acute‐angle takeoff, intramural course, and interarterial trajectory, which have been associated with ischemia, myocardial infarction, and sudden cardiac death [10].

Patients with AOCAOS can be asymptomatic or have a range of clinical presentations, from MI, angina and cardiomyopathy to SCD. In addition, after hypertrophic cardiomyopathy, AOCAOS is considered the second cause of SCD in young patients. The risk of SCD increases with specific pathologies such as interarterial courses, ostial tightness, acute take‐off angle, or intramural path [2].

In symptomatic patients, ECG findings are nonspecific, varying from ST elevation MI to VT or atrial fibrillation [5]. TTE has a role for detecting these anomalies in children. Still, it is operator‐dependent in adults due to the low precision required to determine the type of abnormality, the coronary ostium and the course of the vessels [2]. ICA has high sensitivity and specificity [1] and can evaluate high‐risk anatomical features such as narrowing of anomalous coronary artery [5], but is not able to precisely visualize other structures and we should also consider that it is an invasive procedure [1]. Coronary computed tomography angiography (CCTA) is currently regarded as the gold standard modality due to its fast and clear results, which can delineate the origins and full paths of coronary anomalies [5]. In anatomically complex cases like this, functional imaging such as stress CMR or FFR‐CT could provide valuable data on ischemia burden to further guide management, though it was not utilized here because they were not available.

Although there are no guidelines for appropriate treatment in patients with transseptal course [7], we should individualize our decision for each patient based on risk assessments and symptom burden [5]. Patients with higher risk features such as interarterial and intramural courses and who have had aborted SCD, evidence of myocardial ischemia, syncope or chest pain should be considered for surgical operations [1, 3] such as coronary artery bypass graft, reimplantation of the coronary ostium and unroofing methods with restoration of normal anatomy and percutaneous treatment [2].

Several cases with AOCAOS have been reported so far in the literature but just a few cases were associated with DCM. We know that CAA can cause cardiomyopathies [11]. DCM is defined as dilated one or both ventricles that are not functional. These patients present with clinical manifestations of heart failure such as dyspnea, congestive edema (as well as our patient) and orthopnea. Another dangerous presentation is arrhythmia. Both atrial and ventricular arrhythmias are included. For the initial workup, an echocardiography and ECG should be done first. We can see ventricular dilation and diffuse hypokinesia in echocardiography, which we detected in our patient's echocardiography, too. ECG can demonstrate pathologic Q waves, atrial fibrillation, VT, LBBB (as in our case) and hemiblocks. CMR is the best choice for determining the etiology. In addition, we can also rule out possible ischemic etiologies due to CAD by ICA. Lastly, for idiopathic DCM, it is recommended to do a genetic test, which can influence our treatment because some mutations respond poorly to our medications, and we should change the drugs. In our case, genetic tests were not performed due to financial constraints and lack of access to specialized genetic counseling services. For treatment, our goal is to manage heart failure and arrhythmias. Diuretics, vasodilators, aldosterone receptor blockers, angiotensin receptor neprilysin inhibitors and beta blockers can be used for heart failure. For patients with a history of an episode of VT who could rescue from or have symptomatic VT or in post‐ischemic DCM for primary prevention, we can use implantable cardioverter defibrillators (ICD) [12].

To our best knowledge, including our case, only seven case reports have been reported of AOCAOS with DCM ([2, 13, 14, 15, 16] (Table 1) and Barrios Alonso et al. case report (which we couldn't find the whole text) [17]) since 1990. Our case contributes to this limited literature by demonstrating the value of a comprehensive multimodality imaging approach (ICA, CMR, CCTA) and reporting successful management with CRT‐D implantation. The potential pathophysiological link between AOCAOS and DCM remains unclear. Possible mechanisms include chronic myocardial ischemia due to dynamic compression of the anomalous coronary artery during systole, leading to repetitive stunning and eventual ventricular remodeling. Alternatively, there might be a shared genetic predisposition for both congenital coronary anomalies and cardiomyopathy. However, no definitive correlations have been found between AOCAOS and DCM. Further investigations are needed to determine whether genetic mutations are associated with this anomaly or whether the anomalous artery leads to DCM by itself through a special mechanism.

**TABLE 1:** Current reported cases of anomalous origin of coronary artery associated with DCM.

Case	Age/sex	Anomaly	Clinical presentation	Genetic test	Treatment and follow‐up
Mrad et al. [1]	63/male	The common trunk of LMCA^a^ and RCA^b^ originated from RCC^c^	Heart failure	No test was done	Stent implantation and medical therapy/Remain asymptomatic
Hernandez et al. [13]	49/female	The RCA^b^ originated from the left coronary cusp	Palpitation and light‐headedness	BAG3 mutation‐positive	Surgery and medical therapy/except VT^d^, other symptoms resolved
Orr et al. [14]	10/female	LMCA^a^ originated from RCC^c^	Exertional and non‐exertional chest pain	SMAD6 gene positive	Percutaneous coronary intervention and medical therapy/remain asymptomatic
Attar et al. [15]	65/female	RCA^b^ originated from the proximal part of the LAD^e^ artery	Heart failure	No test was done	Medical therapy
Okura et al. [16]	64/male	LAD^e^ originated from the right coronary cusp	Heart failure	No test was done	No further information

^a^ Left main coronary arter.

^b^ Right coronary artery.

^c^ Right coronary cusp.

^d^ Ventricular tachycardia.

^e^ Left anterior descending.

A comparison of management strategies reveals a distinct therapeutic approach in our case relative to those previously reported. As summarized in the table, interventions for similar anomalies have primarily focused on directly addressing the coronary anatomy itself, employing modalities such as stent implantation (Mrad et al. [2], Orr et al. [14]) or surgical correction (Hernandez et al. [13]), often alongside medical therapy for heart failure. In contrast, for our patient with a transseptal anomalous left main coronary artery and severe underlying dilated cardiomyopathy, the heart team elected for a device‐centric strategy focused on managing the resultant cardiomyopathy and preventing sudden cardiac death. This involved optimal medical therapy combined with Cardiac Resynchronization Therapy Defibrillator (CRT‐D) implantation, rather than a direct coronary intervention. This approach underscores that when a coronary anomaly is associated with significant ventricular dysfunction and life‐threatening arrhythmias, the primary management priority may shift towards device‐based heart failure and arrhythmia management, particularly when the anatomical variant is not considered high‐risk for ischemia and there is no definitive evidence of ischemia directly attributable to the anomaly.

Another unexplained matter is the DCM detected by CMR. The absence of late gadolinium enhancement (LGE) on CMR in our patient is noteworthy. It is critical to state that the LGE technique is exquisitely sensitive for detecting focal, replacement fibrosis (where collagen replaces dead myocytes). However, it is relatively insensitive to diffuse, interstitial fibrosis, which can be present in various cardiomyopathies. The absence of LGE does not rule out the presence of any fibrosis in this patient [18]. Also there may be another explanation for that: chronic repetitive ischemia because of “Anomalous Left Main Coronary Artery from Right Coronary Cusp with Transseptal Course” can result in myocardial stunning and hibernation, which can manifest as LV dilation and dysfunction—a DCM phenotype. Crucially, chronic ischemia from this mechanism typically does not cause replacement fibrosis (which is what LGE detects) until its very late, end stage. Instead, it causes apoptosis and myocyte loss without a significant collagenous scar [19]. The severely reduced EF in the absence of fibrosis raises the possibility of a primary myocardial disorder (e.g., a genetic mutation affecting sarcomeric proteins) where ventricular dysfunction precedes overt fibrotic change, or microvascular dysfunction not detectable by conventional LGE imaging.

### 5.1 Limitations

This report has several limitations. It is a single case, limiting generalizability. The absence of genetic testing and endomyocardial biopsy means we cannot definitively rule out a genetic or inflammatory etiology for the DCM. Furthermore, functional ischemia assessment (e.g., with FFR or stress imaging) was not performed. A 3D reconstruction of the CCTA images was not available, which would have enhanced anatomical understanding.

## 6 Conclusion

AOCAOS is one of the rarest kinds of CAA. It could be considered a benign or malignant anomaly, which depends on the course of the anomalous coronary artery. Patients with this anomaly present with MI, arrhythmia, and SCD. Although there are no specific guidelines for diagnosing or treating AOCAOS, CCTA is known as the gold standard modality for diagnosis. Management of these patients varies from medical therapy to surgery due to the low or high‐risk course of the anomalous coronary artery. In this case, we report a patient with the anomalous origin of LMCA from RCC with DCM, which is a rare but critical association, and no detailed correlation has been found between these two so far. Further studies are needed to determine whether DCM is an incidental finding or an associated feature. Clinicians should consider coronary anomalies in the differential diagnosis of non‐ischemic DCM, especially in patients presenting with arrhythmias, and employ CMR, coronary CTA and genetic testing for comprehensive assessment.

## Author Contributions

**Amir Heidari:** conceptualization, project administration, supervision, writing – original draft. **Enssieh Hashemi:** investigation, writing – review and editing. **Mehrdad Jafari Fesharaki:** data curation, investigation. **Golnaz Houshmand:** investigation. **Roghaye Ghiasvand‐Mohammadkhani:** data curation, writing – original draft, writing – review and editing.

## Consent

Written informed consent was obtained from the patient to publish this report in accordance with the journal's patient consent policy. It was reviewed and approved with the ethics code IR.SBMU.RETECH.REC.1403.485 at the Vice Chancellery for Research and Technology of Shahid Beheshti University of Medical Sciences.

## Conflicts of Interest

The authors declare no conflicts of interest.

## Data Availability

The data that support the findings of this study are available from the corresponding author upon reasonable request.
